# Exploring the potential benefits of multi‐field IMPT for stage I NSCLC SBRT: An in silico dosimetric comparison to IMRT and CyberKnife

**DOI:** 10.1002/pro6.70027

**Published:** 2025-08-26

**Authors:** ZhangMing Chen, Xianrui Yan, Shu Xie, Pinjing Cheng, Dong Xiang, Cheng Tao, Qingtao Qiu, Tengxiang Li, Chengqiang Li, Huazhong Shu, Antoine Simon, Jian Zhu

**Affiliations:** ^1^ School of Nuclear Science and Technology University of South China Hengyang Hunan Province China; ^2^ Shandong Provincial Key Medical and Health Laboratory of Pediatric Cancer Precision Radiotherapy Shandong Cancer Hospital and Institute, Shandong First Medical University and Shandong Academy of Medical Sciences Jinan Shandong Province China; ^3^ Department of Radiation Oncology Physics Shandong Cancer Hospital and Institute, Shandong First Medical University and Shandong Academy of Medical Sciences Jinan Shandong Province China; ^4^ Centre de Recherche en Information BioMdicale Sino‐ français Nanjing China; ^5^ Centre de Recherche en Information BioMdicale Sino‐français Rennes France

**Keywords:** Dosimetric study, Multi‐field intensity‐modulated proton therapy, Stage I non‐small cell lung cancer, Stereotactic body radiotherapy

## Abstract

**Purpose:**

Multi‐field intensity‐modulated proton therapy (IMPT) is a novel treatment protocol design method proposed to reduce range uncertainty. This study aimed to investigate whether multi‐field IMPT has a dose distribution advantage over photon intensity‐modulated radiation therapy (IMRT) and CyberKnife in stereotactic body radiotherapy (SBRT) for stage I non‐small cell lung cancer (NSCLC).

**Methods:**

Twenty‐nine patients who underwent photon SBRT from February 2021 to September 2022 at Shandong Cancer Hospital were included. Their Computed Tomography (CT) images were used to design CyberKnife and multi‐field IMPT plans. For the photon plan (IMRT and CyberKnife), the planning target volume (PTV), which was extended from the internal gross target volume (IGTV), was prescribed at 50 Gy. For the proton plans, the planning beam‐specific target volume (PBSTV) based on the IGTV was created to meet the same area as the photon PTV. Multi‐field IMPT was simulated by adding additional beam angles to conventional IMPT. Dose distribution assessment factors included D_mean_ and dose gradient index (GI) for PTV/PBSTV, and D_mean_ and the hottest 0.1 cm^3^ dose (D_0.1cc_) for organs at risk (OARs).

**Results:**

With each patient receiving 7–11 beams, multi‐field IMPT had a better target GI than IMRT. For the lung, heart, spinal cord, chest wall, and ribs doses, the D_mean_ of the multi‐field IMPT was smaller than that of the other two plans for all metrics. CyberKnife was significantly less protective of the OARs than the other two planning modalities, owing to the presence of a high target center dose.

**Conclusion:**

Multi‐field IMPT achieves favorable target coverage and OAR protection compared to IMRT and CyberKnife for SBRT of NSCLC.

## Graphical abstract

It is apparent that the dose distribution achieved with the multi‐field IMPT technique exhibits a lower maximum dose than both IMRT and Cyberknife. multi‐field IMPT achieve favorable target coverage and OARs sparing compared to IMRT and Cyberknife for SBRT of NSCLC. For the clinical application of multi‐field IMPT therapy, further research is still needed on aspects such as refining the field of fire angle.

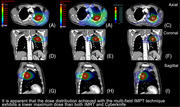

## 1 INTRODUCTION

Despite progress in cancer treatment modalities, global statistics still designate lung cancer as the predominant malignancy, imposing a substantial disease burden worldwide.^1^, ^2^ Furthermore, epidemiological data from European and North American populations have consistently reported lung cancer as the primary etiology contributing to mortality outcomes in these regions.^3^ Non‐small cell lung cancer (NSCLC) accounts for approximately 80% of lung cancer cases and is associated with a poor prognosis, even after aggressive treatment. The integration of stereotactic body radiotherapy (SBRT) into early‐stage NSCLC management has increased radiotherapy utilization and decreased the proportion of untreated patients, contributing to improved population‐level survival.^4^ SBRT is more cost‐effective than lobectomy in the treatment of early‐stage NSCLC.^5^ The results of the RTOG0618 randomized controlled trial^6^ indicate that SBRT provides high rates of local control, results in minimal treatment‐related toxicity, and rarely requires salvage surgery in patients with operable early‐stage NSCLC.

The field of photon‐based SBRT has advanced from three‐dimensional conformal radiotherapy through intensity‐modulated radiation therapy (IMRT) to volumetric‐modulated arc therapy (VMAT).^7^, ^8^ Compared to traditional photon therapy, the physical dosimetry advantage of protons lies in the truncated dose drop at the trailing edge of the Bragg peak.^9^, ^10^ The spatial distribution of Bragg peaks at tumor target locations optimizes the defined radiation absorption within the cancer volume while minimizing unintentional exposure to the surrounding normal tissue.^11^, ^12^, ^13^ By precisely adjusting the co‐localization of the Bragg peak with the target lesion, sharp dose attenuation can be achieved at the tumor‐normal tissue interface. This characteristic of particle therapy allows highly conformal dose deployment to the tumor target while reducing the absorbed dose to the proximal and distal risk organs in patients with lung cancer.^14^ Exploiting the dosimetric benefits of protons provides an opportunity to safely escalate the tumor dose, which may improve clinical treatment outcomes.^12^

Recently, the use of proton radiotherapy for SBRT of lung cancer has been increasingly investigated. Despite the challenges of treating moving lung tumors with protons, such as range uncertainty and interaction effects, preliminary results seem to be more advantageous than those of photon‐based radiotherapy.^15^ Dosimetric analyses of hypofractionated proton therapy for NSCLC demonstrated superior dose distributions and significantly lower doses to the heart and lungs compared with photon techniques. In hypofractionation, proton SBRT may spare normal tissues better than conformal photon techniques.^16^ Additionally, protons allow safe dose escalation to tumor volumes that cannot be achieved with photons, particularly for larger tumors in challenging locations.^17^

Proton radiotherapy techniques have evolved from passive scattering approaches to pencil beam scanning and intensity‐modulated proton therapy (IMPT).^18^ The latest advancement is proton arc therapy (PAT), in which a proton beam is delivered while the gantry rotates continuously around the patient.^19^ Research into the clinical implementation of PAT commenced in 2016.^20^, ^21^ Despite being unavailable in clinical settings, several planning studies have demonstrated the feasibility of PAT and potential dosimetric advantages compared with those of IMPT across multiple disease sites, including head and neck,^22^ brain,^23^ lung,^24^ and prostate cancers.^25^ The reported benefits of PAT include improved dose conformality to the target volume, enhanced robustness against setup errors, beam range uncertainties, organ motion, and no significant prolongation of treatment delivery time compared with those of IMPT.^19^, ^20^, ^21^ Ongoing research aims to translate these dosimetric advantages into clinical gains once PAT is clinically implemented.

Uncertainty is a huge challenge for proton radiotherapy, including uncertainty in the range and uncertainty of the relative biological effectiveness (RBE). For the lungs, range uncertainty is a significant problem because the density of the incident path varies greatly, and CT cannot accurately reflect the tissue density when there is respiratory movement. The solution to the range uncertainty is to use multi‐field, non‐coplanar radiation therapy. Arc therapy is the best method to overcome these uncertainties. Because our hospital does not have a proper proton arc plan optimizer, we simulated the configuration by increasing the beam angle based on traditional proton radiotherapy. Multi‐field was used to further reduce the deposition dose in the proton incident path. Although proton plans have fewer low‐dose regions than photon plans, the dose deposited on the spread‐out Bragg peak proximal path is approximately 60% of the prescribed dose. If there are more incident fields, the dose on the incident path can be further reduced, which significantly reduces the range uncertainty.

The aim of this study was to compare different radiotherapy planning techniques, specifically multi‐field IMPT, IMRT, and CyberKnife, in a cohort of stage I NSCLC patients receiving radical SBRT, in order to investigate whether multi‐field IMPT offers a dosimetric advantage over IMRT and CyberKnife in terms of target dose distribution, integral dose, and OARs dose.

## 2 MATERIALS AND METHODS

### 2.1 Patient selection

This dosimetric study retrospectively evaluated 29 patients with early‐stage NSCLC who were treated with SBRT at Shandong Cancer Hospital between February 2021 and September 2022. The cohort included 22 males and 7 females, with a median age of 72 years (range 43–81 years). All patients had unresected T1‐T3N0M0 (stage I/II) NSCLC and were enrolled in the multicenter PACIFIC‐4/RTOG‐3515 clinical trial.

### 2.2 Target and organs at risk (OARs) definition

Target volume and OAR delineation for all 29 patients was performed at Shandong Cancer Hospital following institutional guidelines. Four‐dimensional CT (4DCT) simulations were acquired using a Siemens dual‐source CT (Siemens SOMATOM Definition, DER) under deep inspiration breath‐hold. OARs, gross tumor volume (GTV), clinical target volume (CTV), and planning target volume (PTV) were contoured on the 4DCT images.

The CTV is considered equivalent to the GTV with no prophylactic margin expansion during lung SBRT. The GTV encompasses only abnormal radiographic signals, consistent with the primary tumor bulk (i.e., GTV = CTV). The internal gross target volume (IGTV) accounts for the GTV motion. The IGTV outlines the spatial boundaries encompassing the GTV as it maneuvers throughout the respiration cycle, distinguished from the conventional internal target volume, which encapsulates CTV mobility. The PTV is characterized as the IGTV supplemented with a setup margin to compensate for the uncertainties inherent in treatment delivery. In the current investigation, a standardized 5 mm isotropic expansion was applied to the IGTV contours to generate the PTV, allowing adequate coverage of the tumor path while accounting for potential patient and system geometric variations during treatment. The PTV margin does not include internal motion uncertainty, which is inherently considered in the definition of IGTV. For proton plans, the planning beam‐specific target volume (PBSTV) refers to the proton beam‐specific target volume derived from the IGTV. It is designed to ensure that the irradiated region in multi‐field IMPT treatment plans matches the PTV used in photon therapy or CyberKnife radiosurgery.

Dose constraints were established for the following critical structures that could be affected: the mediastinum (including the heart, trachea, and bronchial structures), chest wall, lungs, esophagus, ribs, and spinal cord. The ribs were contoured within 5 cm around the PTV/PBSTV, excluding intercostal muscle tissue. The chest wall was defined as a 3 cm expanded region originating from the lung tissues. The left and right lungs were individually contoured (Lung‐L and Lung‐R), and subsequently combined to form a single pulmonary structure (Lung). Additionally, the Lung was divided into the ipsilateral lung (Ipsi‐Lung) and contralateral lung (Contra‐Lung) based on the location of the PTV/PBSTV. To assess the total lung dose, the IGTV was subtracted from the total lung volume (Lungs‐IGTV). The IGTV and ipsilateral trachea/bronchus were excluded from the Lungs‐IGTV structure. All the OARs were contoured by a radiation oncologist and reviewed by a second oncologist for quality assurance.

### 2.3 Treatment planning

According to the dosimetric criteria of the multicenter PACIFIC‐4/RTOG‐3515 trial protocol (AstraZeneca), the prescribed dose was standardized to 50 Gy in five fractions. The planning objectives were consistent with several cooperative group trials (e.g., RTOG 0236, 0618, 0813, 0915, and 1106) in which ≥95% of the PTV/PBSTV was to receive ≥100% of the prescribed dose, with the maximum dose (D_max_) not exceeding 140% of the prescription. OAR constraints were as follows: spinal cord ≤28 Gy to 0.03 cm^3^; esophagus ≤35 Gy to 0.03 cm^3^; heart ≤38 Gy to 0.03 cm^3^; brachial plexus ≤32.5 Gy to 0.03 cm^3^; and ribs ≤57 Gy to 0.03 cm^3^.

### 2.4 Multi‐Field IMPT

Multi‐field IMPT was generated in Eclipse v15.6 (Varian Medical Systems, Palo Alto, CA) with a RBE of 1.1 for proton dose calculation. For simplicity, proton Gy (RBE) is presented as Gy. Beam angles were selected to minimize the range uncertainty. Concerning the IMPT field direction and based on the characteristics of the proton plan and clinical treatment experience, the beam directions with large changes in HU values on the incident path were deleted (e.g., traversing air cavities or bone interfaces). The PBSTV margins incorporated a range uncertainty of 3.5% plus 5 mm for the setup error and respiratory motion. Robust optimization was employed using range shift to account for uncertainties.

Because a proper proton arc plan optimizer was not available, multi‐field techniques were used to approximate the arc delivery. Couch rotations enable beam directions to avoid critical structures when necessary. Robustly optimized multi‐field IMPT was generated using a single‐field optimization method in the treatment planning system.

Robust plan evaluation was added to the multi‐field IMPT: plan uncertainty parameters were generated during dose calculation; an isocenter shift of 0.50 cm was added in the X, Y, and Z directions, respectively; and the calibration curve error was 3.5%. The following were generated: U1 X: +0.50 cm + 3.50%, U2 X: +0.50 cm ‐3.50%, U3 X: ‐0.50 cm +3.50%, U4 3.50%, U7 Y:‐0.50 cm +3.50%, U8 Y: ‐0.50 cm ‐3.50%, U9 Z: +0.50 cm +3.50%, U10 Z: +0.50 cm ‐3.50%, U11 Z:‐0.50 cm + 3.50%, and U12 Z: ‐0.50 cm ‐3.50%.

### 2.5 Photons (IMRT & CyberKnife)

IMRT plans were generated using Eclipse v15.6 (Varian Medical Systems, Palo Alto, CA, USA). Seven to eleven 6MV photon beams were utilized while avoiding entrance through the Contra‐Lung. For central lesions, the beam angles were optimized based on the proximity to critical structures. Noncoplanar beam arrangements were employed for IMRT.

CyberKnife plans were created using Precision v1.1 (Accuray Inc., Sunnyvale, CA, USA). Because this was a dosimetric comparison study within a treatment planning system, fiducial implantation and tracking were not performed. All CyberKnife plans used the Body multi‐leaf collimator treatment anatomy, full path set, and Xsight Spine tracking method. For lesions near the arms, beam angles were set to exit only.^26^

### 2.6 Beam arrangements

**FIGURE 1:**
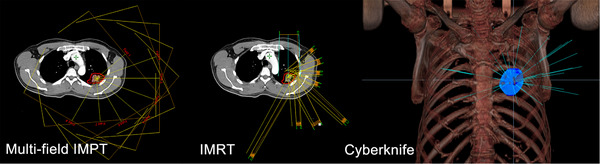
Schematic diagram of beam arrangements for radiotherapy planning techniques in a representative patient. Shown from left to right are 9‐field non‐coplanar multi‐field IMPT beam angles, 9‐field non‐coplanar IMRT plan beam angles, and the CyberKnife plan showing beam angles. IMPT, intensity‐modulated proton therapy; IMRT, intensity‐modulated radiation therapy.

Examples of beam arrangements for radiotherapy planning techniques are shown in Figure 1.

### 2.7 Statistical analysis

Dosimetric parameters were evaluated and compared between plans for the PTV/PBSTV and OARs. For PTV/PBSTV, D_mean_ was assessed. The conventional gradient index (GI) and a proposed dose gradient index (DGI) were calculated to quantify and compare the dose fall‐off from the target to the surrounding OARs. The GI quantifies the ratio of the 50% prescription isodose volume to the PTV/PBSTV, whereas the DGI characterizes the ratio of prescription isodose volumes to the PTV/PBSTV evaluated over a range of dose percentages. Therefore, while the GI provides a single metric of dose fall‐off at half the prescribed level, the DGI more comprehensively depicts the dose gradient behavior from the planning aim point through lower levels encompassed by the target definition.

(1) $$DGI=100-\left\{100\times \left(\left(R_{eff,\;50\%R_{x}}-R_{eff,\;R_{x}}\right)-0.3cm\right)\right\}$$

(2) $$\;R_{eff}=\sqrt[{3}]{\frac{3V}{4\pi }}\;$$

(3) $$GI=\frac{R_{eff,\;R_{x}}}{R_{eff,\;50\%R_{x}}}$$

The DGI was computed according to Equation 1, where R_eff_ is the effective radius of the prescription isodose (R_x_) volume and R_eff,50%Rx_ is the effective radius of the 50% R_x_ isodose surface.^27^, ^28^, ^29^, ^30^ The effective radius of a specified volume is mathematically defined as the spherical radius that yields an equivalent volume, as defined by Equation 2, where V is the isodose surface volume. The GI quantifies the dose fall‐off gradient by comparing the effective radii of the prescription isodose volumes to the 50% prescription dose level (Equation 3).

This study involved 29 patients who were divided into three groups (A, B, and C) based on the location of the target region within the lungs. Group A included patients with target areas proximal to the chest wall, group B included those proximal to the mediastinum, and group C included those in the central lungs.

The doses to different lung volumes were recorded, including D_mean_, V_5Gy_, V_20Gy_, V_35Gy,_ V_40Gy_, and V_50Gy_. For other OARs, the dose to the hottest 0.1 cm^3^ (D_0.1cc_) was compared as a surrogate for the maximum dose. For the heart, V_5Gy_ and V_30Gy_ were also evaluated.

Statistical analyses were performed using SPSS v26.0 (SPSS Inc., Chicago, IL, USA). Given the sample size and data distribution, nonparametric tests were used for all analyses. The Wilcoxon signed‐rank test was used to compare dosimetric parameters. A *P*‐value of < 0.05 was considered statistically significant. The *P*‐value was corrected using the Benjamini‐Hochberg method. A nonparametric methodology was preferred over parametric tests because of the limited sample size.

## 3 RESULTS

**FIGURE 2:**
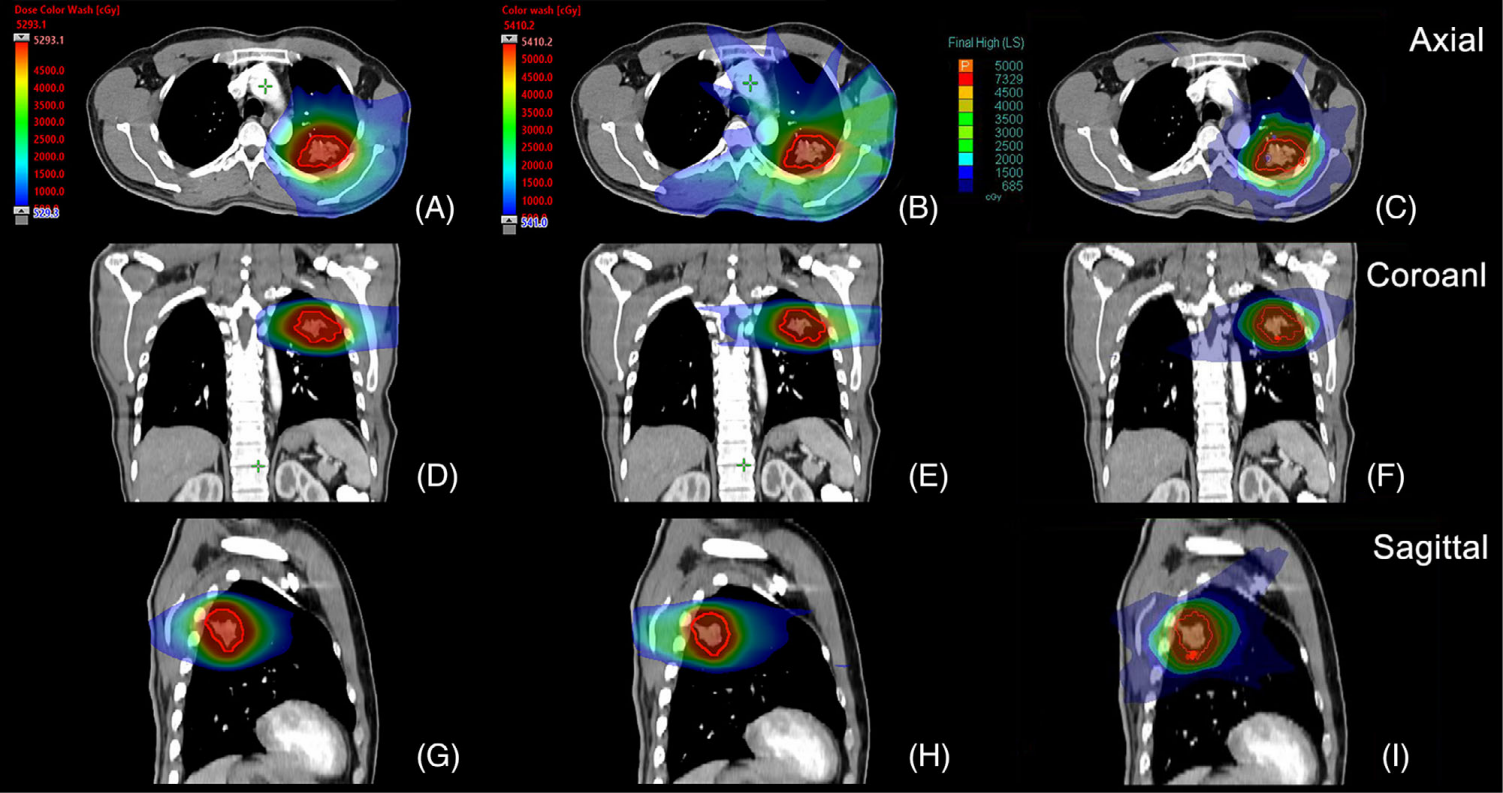
Dose distribution comparison in axial, coronal, and sagittal planes for radiotherapy planning in a representative patient. This diagram shows the (a, d, g) 9‐field non‐coplanar multi‐field IMPT, (b, e, h) 9‐field non‐coplanar IMRT, and (c, f, i) CyberKnife plans. IMPT, intensity‐modulated proton therapy; IMRT, intensity‐modulated radiation therapy.

**FIGURE 3:**
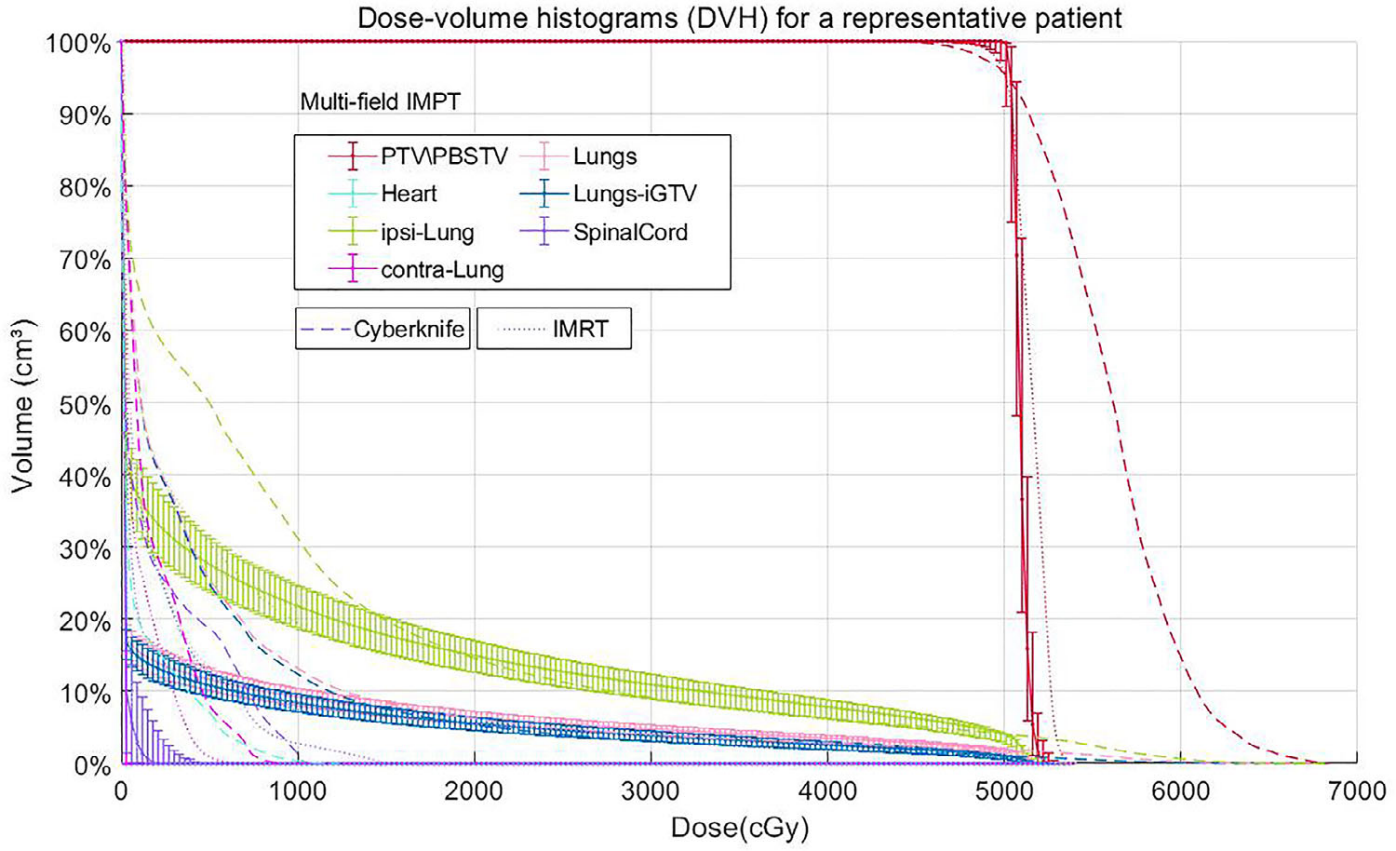
Dose‐volume histograms (DVHs) for the three plan designs for a representative patient. Robust plan evaluation of multi‐field IMPT is represented by error bars.IMPT, intensity‐modulated proton therapy; IMRT, intensity‐modulated radiation therapy; PBSTV, planning beam‐specific target volume; PTV, planning target volume; IGTV, internal gross target volume.

The mean PTV was 48.4 cm^3^ (range 10.1‐161.7 cm^3^). The PTV/PBSTV dose distributions for a typical case are shown in Figure 2. The PTV/PBSTV coverage criterion of V_95%_ > 100% of the prescribed dose was achieved in all radiotherapy plans for both the nominal plans and the uncertainty scenarios generated for robustness evaluation (Figure 3). Nominal plans represent idealized treatment scenarios without accounting for delivery uncertainties (e.g., respiratory motion and setup errors). Uncertainty scenarios were generated by simulating organ displacements (e.g., ±3 mm) and dose calculation errors to evaluate plan robustness. All plans (including worst‐case scenarios) maintained V_95%_ >100%, demonstrating that the target dose coverage satisfied the clinical requirements even under extreme conditions (e.g., organ motion or machine inaccuracies).

Multi‐field IMPT resulted in a lower mean PTV/PBSTV dose than IMRT (5053 cGy vs. 5243 cGy, *P* < 0.01). When the plan requirements were met, the closer the average dose was to 5000 cGy, the fewer were the high‐dose areas within the target area, indicating better dose uniformity in the target area. The mean and maximum target doses were significantly higher for CyberKnife than for IMRT and multi‐field IMPT (*P* < 0.01 for both modalities). Similar dose characteristics were observed for the IGTV, with multi‐field IMPT and IMRT having a significantly lower mean IGTV dose than CyberKnife (5205 cGy vs. 5308 cGy vs. 6007 cGy, *P* < 0.01).

The dose distribution achieved with the multi‐field IMPT technique exhibited a lower maximum dose than that with both IMRT and CyberKnife (Figure 2). Our findings suggest that constrained maximum dose optimization, subject to maintaining the prescribed target dose requirements, contributes to reduced acute toxicity profiles. Additionally, by minimizing low‐dose leakage beyond the PTV/PBSTV, multi‐field IMPT at prescribed doses substantially decreases OAR toxicity risks. This rapid dose fall‐off of the multi‐field IMPT outside the PTV/PBSTV was further evidenced in dose‐volume histograms (DVHs) (Figure 3). The 12 uncertainty curves were represented using error bars after exporting the data from the planning system.

Multi‐field IMPT achieved a steeper dose gradient beyond the PTV/PBSTV. Regarding OARs, the DVH revealed that multi‐field IMPT could also achieve lower dose values with respect to dose metrics than those achievable with IMRT or CyberKnife. The superior dose fall‐off characteristics of multi‐field IMPT restrict low‐dose spread to extra‐target regions at therapeutic dose levels, resulting in reduced radiation damage to OARs.

A robust evaluation of the treatment plan was performed using DVH analysis. The DVH plots for the 12 perturbed dose distributions using error bars are shown in Figure 3. The DVH curves of the perturbed dose distributions were compared with the DVH curves of the initial plan, represented by solid lines. Notably, the DVH curves of the target and OARs showed a high degree of similarity, suggesting a comparable robust plan quality. In the multi‐field IMPT, perturbed dose distributions for all OARs in the DVH were superior to those in IMRT and CyberKnife.

### 3.1 Differences in the DGI

**FIGURE 4:**
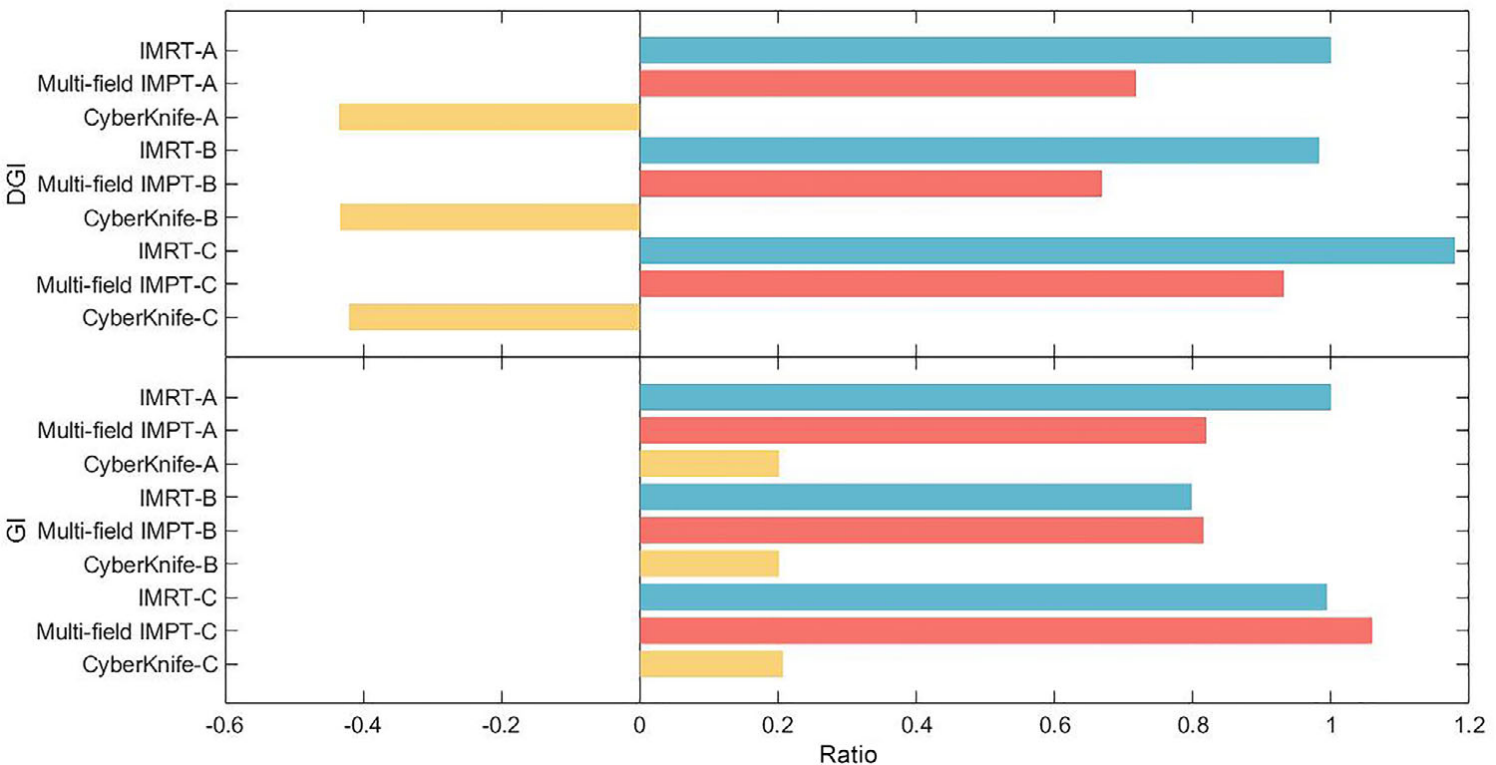
Normalized data plot with IMRT‐A as a reference. The normalized gradient index (GI) and dose gradient index (DGI) values obtained from treatment planning simulations are depicted using three different modalities: multi‐field IMPT, IMRT, and CyberKnife. Normalization was performed relative to the GI and DGI values from IMRT‐A planning. The smaller the normalized GI and DGI indices are indicative of more favorable dose fall‐off characteristics. IGTV, internal gross target volume; IMRT, intensity‐modulated radiation therapy.

The values obtained from the DGI and conventional GI for IMRT‐A were normalized to 1 for comparative purposes. From Equations (1), (2), (3), lower normalized GI and DGI values express superior dose fall‐off characteristics (Figure 4).

Plans for group C, patients with central lung tumors, yielded the highest normalized GI versus DGI values across all three treatment modalities, showing the poorest dose fall‐off effects in this cohort. CyberKnife resulted in significantly less optimal sparing of organs at risk than the other modalities (Figure 3). However, CyberKnife had a lower GI and DGI than IMRT and multi‐field IMPT in the three groups, which maintained the most favorable relative dose fall‐off.

Trends in normalized GI and DGI were broadly consistent, with multi‐field IMPT plans exhibiting improved dose fall‐off relative to IMRT, particularly for the DGI, which was more pronounced (0.7372 vs. 1, *P* < 0.01).

### 3.2 Dosimetry difference in the lung

**FIGURE 5:**
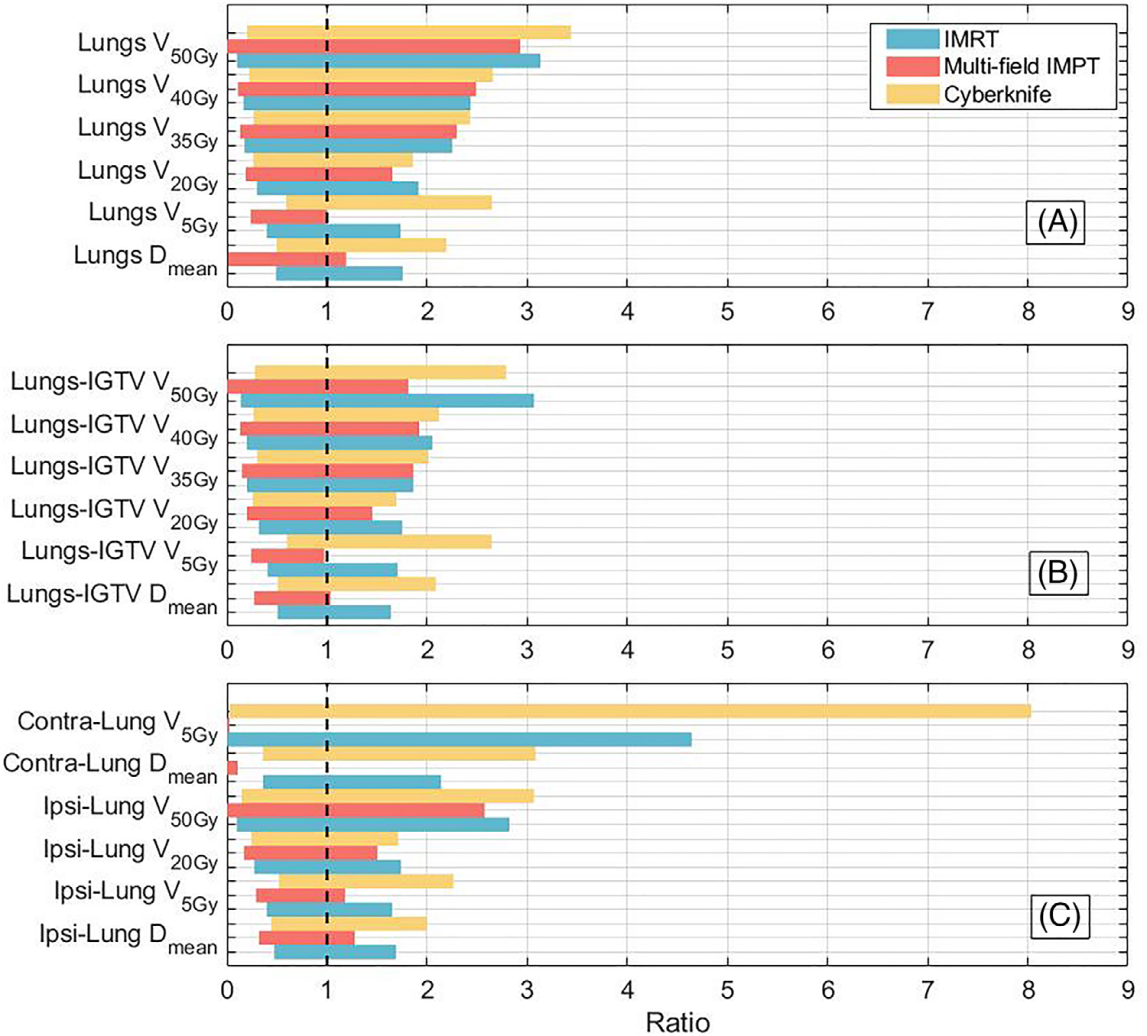
Normalized rectangular plot with D_mean_ for IMRT plans set to 1 and displayed as a black dashed line. Rectangular areas represent a) Lungs, b) Lungs‐IGTV, and c) Ipsi‐Lung and Contra‐Lung. More left‐positioned rectangles indicate smaller D_mean_ values than those of IMRT. D_mean_ = mean dose, V_xGy_ = percentage of the volume receiving xGy or more. Lungs‐IGTV = lungs excluding IGTV, ipsi = ipsilateral, contra = contralateral. Ipsilateral refers to the same side as the IGTV, and contralateral is the opposite side. IGTV, internal gross target volume; IMRT, intensity‐modulated radiation therapy.

While all modalities were able to achieve predefined OAR objectives for most patients when equivalent target doses were prescribed, the analysis of pulmonary parameters revealed differences between the techniques. The mean lung dose from multi‐field IMPT plans was significantly lower than that from IMRT and CyberKnife (Figure 5). For the lungs D_mean_, although the mean absolute difference was not significant, the relative difference values for multi‐field IMPT versus IMRT and CyberKnife were 42.41% and 47.25%, respectively (*P*< 0.01 for both modalities). Regarding the V_5Gy_ parameter representative of low‐dose lung volume, multi‐field IMPT conferred advantages over photon modalities, with 39.94% (*P* < 0.01) and 54.90% (*P* < 0.01) reductions versus IMRT and CyberKnife, respectively.

Multi‐field IMPT demonstrated significantly enhanced organ sparing compared to IMRT and CyberKnife. In terms of Lungs‐IGTV, the D_mean_ and V_5Gy_ of multi‐field IMPT were reduced by 42.23% (*P* < 0.01) and 40.45% (*P* < 0.01), respectively, compared with those of IMRT. For the high‐dose V_50Gy_ parameter of Lungs‐IGTV, multi‐field IMPT yielded 34.13% (*P* < 0.01) and 41.95% (*P* < 0.01) lower relative doses than IMRT and CyberKnife, respectively. Multi‐field IMPT conferred the lowest mean V_5Gy_ for the Contra‐Lung (0.0069%) versus 3.92% for IMRT and 6.96% for CyberKnife. Quantitative analysis of the Contra‐Lung D_mean_ revealed that the multi‐field IMPT plan resulted in a 99.12% lower value than IMRT (*P* < 0.01) and a 99.27% lower value than CyberKnife (*P* < 0.01). In Figure 5, more left‐positioned rectangles indicate smaller D_mean_ values than those of IMRT. Larger rectangular areas below 1 suggest that more values are lower than the standard, representing a more favorable dose value. The upper and lower bounds of the multi‐field IMPT dose distribution curve lie below those of the other two techniques, indicating a lower minimum and maximum dose spillover to the Ipsi‐Lung tissue with multi‐field IMPT.

### 3.3 Dosimetry difference on the heart and other OARs

**FIGURE 6:**
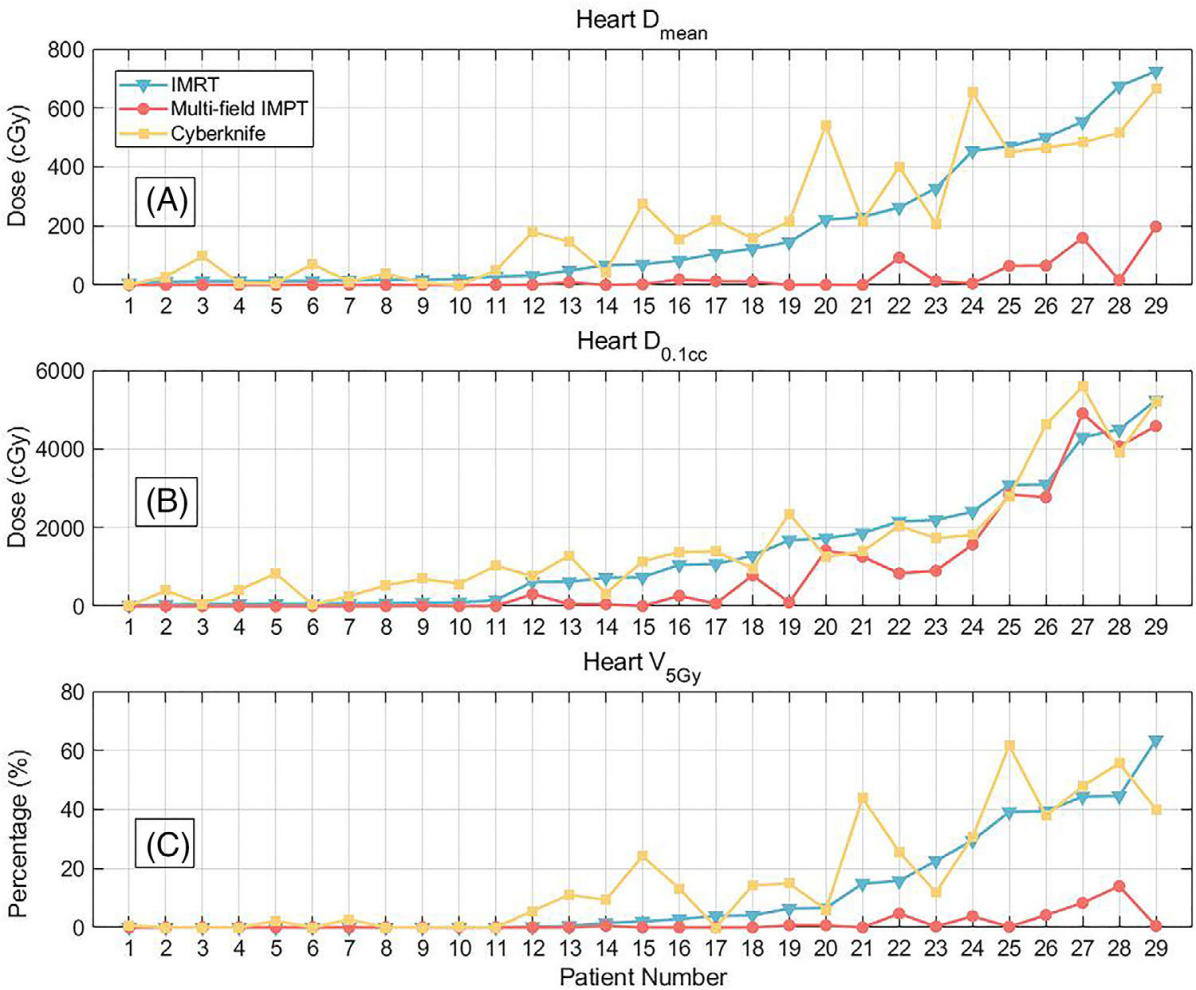
Plots comparing heart dosimetric parameters achieved via multi‐field IMPT, IMRT, and CyberKnife for a representative patient. Metrics included heart a) D_mean_, b) D_0.1cc_, and c) V_5Gy_, sorted by lowest to highest IMRT dose. D_mean_ = mean dose, D_0.1cc_ = dose to the ‘hottest’ 0.1cc, V_5Gy_ = percentage of the volume receiving 5 Gy or more.

Multi‐field IMPT achieved a significantly lower D_mean_ to the heart (23.1 cGy vs. 220.09 cGy, *P* < 0.01), spinal cord (36.56 cGy vs. 150.06 cGy, *P* < 0.01), chest wall (302.76 cGy vs. 614.40c Gy, *P* < 0.01), and ribs (531.83 cGy vs. 941.05 cGy, *P* < 0.01)) than IMRT. The mean doses from CyberKnife were generally higher (Figure 3). Notably, the heart D_mean_ substantially decreased by 87.26% for multi‐field IMPT versus IMRT (*P* < 0.01). The D_0.1cc_ was 89.37% lower with multi‐field IMPT (*P* < 0.01). By heart dosimetry in all 29 patients, multi‐field IMPT had a significantly lower heart D_mean_ and V_5Gy_ than IMRT with CyberKnife (Figure 6). The heart D_mean_, D_0.1cc_, and V_5Gy_ values are shown in Figure 6. sorted according to the lowest to highest IMRT doses.

Across all patients, multi‐field IMPT consistently provided substantial reductions compared to IMRT and CyberKnife for both D_mean_ and V_5Gy_, as evidenced by data points systematically below the others in graphs a) and c), respectively. While high‐dose regions as per D_0.1cc_ seemed comparable among techniques based on graph b), multi‐field IMPT conferred clear advantages regarding heart exposure by lowering D_mean_ and V_5Gy_.

## 4 DISCUSSION

Many dose comparison studies between proton therapy and other modalities have been published in recent years, including comparisons between (1) protons and 3D‐CRT, (2) protons and IMRT, (3) protons and VMAT, (4) protons and CyberKnife, and (5) traditional proton therapy and multi‐field proton therapy.^31^, ^32^, ^33^, ^34^ Previous reports have highlighted the dosimetric advantages of protons, further enhancements of pencil beam technology over passive scattering, and the noticeable dosimetric benefit of multi‐field protons over traditional protons. However, previous studies have not simultaneously simulated and directly compared multi‐field IMPT optimization using pencil beam scanning, IMRT, and CyberKnife technology. Therefore, our study aimed to perform a dosimetry analysis to elucidate the potential advantages of multi‐field IMPT treatment in SBRT for early‐stage NSCLC. These findings substantiate proton therapy, particularly multi‐field IMPT, as a clinically viable option that enhances normal tissue sparing compared to conventional photon IMRT and emerging hypofractionated CyberKnife techniques. This supports the broader clinical adoption of multi‐field IMPT across diverse treatment sites and indications, while providing practical guidance for institutions lacking dedicated proton arc optimization systems.

### 4.1 Dosimetric differences in target coverage across treatment plans

In this study, we found that CyberKnife provided the best dose reduction because of higher prescribed doses to centrally located targets.^29^ CyberKnife normalizes the prescribed dose to the 75% isodose curve; therefore, the dose at the center of the tumor is much higher than that at the edge of the target area, which means that a hot spot in the center of the target area is allowed to exist. In such cases, special attention should be paid to controlling the dose to hotspots on the chest wall and ribs.

As illustrated in Figure 2, multi‐field IMPT demonstrated superior dosimetric characteristics in terms of dose gradient and fall‐off compared to IMRT. The inherent Bragg peak phenomenon enables a steeper post‐target dose reduction than that in photon therapy. Furthermore, when employing fewer daily fields (2‐3 fields), IMPT maintains significantly smaller low‐dose regions. Notably, even when the field number is increased to match the photon techniques (7‐11 fields), proton therapy still achieves superior dose confinement, confirming the homogeneous target coverage characteristic of multi‐field IMPT.

As demonstrated in Figure 4, achieving optimal dose gradients proved more technically challenging in regions surrounded by low‐density tissue (C) than in areas adjacent to higher‐density structures (A and B) across all three planning techniques. Low‐density tissues close to the target require more advanced optimization and calculations to properly sculpt isodose lines and minimize the dose effects on adjacent normal tissues. These results highlight that greater heterogeneity in the peritarget tissue density correlates with increased difficulty in achieving highly conformal dose distributions.

### 4.2 Correlation between beam incidence angles and lung dose distribution

At our institution, the typical approach to proton beam therapy for lung cancer is to select posterior and posterior oblique beam angles. The purpose of the beam configuration is to reduce the uncertainty in the beam path and limit the dose to the lung tissue that passes through. Unlike the low‐dose scattering profile obtained with photon therapy, the density of healthy lung tissue traversed by the proton beam is exposed to a concentrated higher dose within a limited penetration path.^35^

When designing radiotherapy plans for lung cancer, limiting the Ipsi‐Lung exposure to high doses emanating from the target is a key concern for medical physicists.^36^, ^37^ Harris et al. found that, in addition to the commonly used D_mean_ and V_20Gy_, V_35Gy_, V_40Gy_, and V_50Gy_ may predict proton patient outcomes.^38^ Our study also found that multi‐field IMPT has obvious advantages over V_50Gy_. Ran et al. reported that both high‐dose hotspots encompassing small volumes and low‐dose regions incorporating larger volumes were associated with an increased likelihood of radiation‐induced pneumonitis development.^39^, ^40^ Our study revealed that the analysis of dose parameters using a multi‐field IMPT plan provided a more favorable low‐dose volume profile for the Ipsi‐Lung than did IMRT. Owing to the extensive non‐coplanar irradiation of CyberKnife, V_5Gy_ was larger than that of photon SBRT.

During SBRT in patients with lung cancer, multi‐field IMPT may be an advantageous radiotherapy approach to limit radiation exposure to the surrounding normal tissue.

### 4.3 Association between heart radiation dose and cardiotoxicity incidence

All three radiotherapy regimens effectively protected most normal tissues. Owing to the relatively distant location of the tumor, no treatment regimen exceeded the prescribed dose limits. The results of our treatment planning study showed that the heart D_mean_ and V_5Gy_ were the lowest when using multi‐field IMPT, which is consistent with the conclusion of Krista et al.^41^ According to previous reports, lower heart irradiation is associated with a reduced risk of long‐term cardiovascular toxicity.^42^, ^43^ By significantly reducing heart irradiation, multi‐field IMPT may help reduce the likelihood of increased heart morbidity. Our study demonstrates the dosimetric superiority of multi‐field IMPT over alternative modalities for cardiac dose reduction. Given the significant sparing of heart tissue, the clinical implementation of multi‐field IMPT may mitigate radiation‐induced cardiotoxicity, thereby reducing long‐term cardiovascular morbidity risks.

### 4.4 Strengths and innovations of our research

Our study utilized multi‐field IMPT to evaluate dosimetric parameters in high‐dose regions within the irradiated lung and heart structures. The purpose of the quantitative assessment of maximum, mean, and low‐volume doses was to optimize the protection of critical mediastinal organs and reduce the probability of toxicity associated with inadvertent irradiation of the heart or lungs. The next step was to correlate the dosimetric results with clinical endpoints to help verify whether the multi‐field IMPT program could reduce the risk of radiation‐induced morbidity in lung cancer patients. The pioneering in silico study by Krista et al.^41^ presented the largest investigation to date comparing photon‐based stereotactic radiotherapy plans (IMRT, VMAT, and CyberKnife) with particle therapy plans (double scattered proton and intensity‐modulated carbon‐ion) for NSCLC. The study evaluated dosimetric outcomes, specifically doses to OARs and CTV homogeneity. The results demonstrated that particle therapy plans achieved lower OAR doses and more homogeneous CTV doses than photon‐based plans. This result is comparable to that of our study; however, this study used DSP for comparison, and it may be possible to further reduce D_max_ and other OAR doses using pencil beam scanning and IMPT, which was confirmed in our study.

Argota‐Perez et al.^34^ compared the delivery time, dosimetric differences, and robustness of the target coverage of conventional proton field configurations and multi‐field pseudo‐arc configurations. The 11‐field pseudo‐arc configuration provided significant dosimetric benefits to the ipsilateral OAR compared to the traditional IMPT plan, which was the same as the pre‐experimental conclusion of our study and provided the basis for our study. de Jong et al.^44^ showed that, compared with IMPT plans, proton arc plans achieved robust target coverage with a lower dose to healthy tissue and reduced overall dose burden because the integrated dose was reduced by 21% ± 3%, thereby reducing patient risk. Compared to the VMAT plan, normal tissue complication probability (NTCP) was reduced. A comparison of NTCP will be our next research goal.

### 4.5 Several limitations

One limitation of current treatment planning research is that continuous arcs cannot be fully optimized owing to algorithm limitations. However, while 7–11 field plans differ from true arc therapy, they provide insights into the potential advantages of this technology in terms of the robustness of target coverage and dose distribution to OAR. With ongoing optimization and clinical validation, multi‐field IMPT may emerge as a viable alternative to conventional 2 and 3‐field proton therapy, demonstrating superior organ‐at‐risk sparing for specific anatomical sites and clinical indications.

Another limitation of the current study was the lack of modeling and comparison of tumor control probability (TCP) and NTCP between treatment techniques. However, as plans are generated based on clinical dose standards to ensure equivalent target coverage, the physical dosimetry parameters representing OAR exposure still have a certain reference value. Future work will prospectively report clinical outcomes related to TCP and NTCP values to verify whether the protection against OAR observed with multi‐field IMPT programs can be empirically translated into reduced toxicity rates and improvements in patient‐reported quality of life.

Another limitation is that the study does not consider changes in the RBE of protons above 6 Gy in a single dose. Previous reports have shown^45^ that the effect of RBE1.1 may be reduced in this case; however, there are currently no studies showing the specific reduction value. Therefore, relevant prospective clinical studies are required to determine whether the dosimetric advantages shown by the multi‐field IMPT program in silico dose comparison can bring meaningful therapeutic effects to patients.

## 5 CONCLUSIONS

Employing 7–11 field plans in our methodology minimized the doses to the lungs and heart while maintaining a clinically acceptable prescription dosage of the target. Multi‐field IMPT was able to produce plans with a reduced integral dose, OAR dose, and plan toxicity when compared to IMRT and CyberKnife for Stage I NSCLC patients. Multi‐field IMPT may be a novel radiotherapy planning approach that combines the potential benefits of the proton Bragg peak and a lower proximal planner dose, which can provide a new treatment planning method for institutions without proton arc plan optimizers.

## CONFLICT OF INTEREST STATEMENT

The authors declare no conflicts of interest.

## ETHICS STATEMENT

This study was approved by the Research Ethics Committee of Shandong Cancer Hospital and Institute, and written informed consent was obtained from all participants.

## Data Availability

The data supporting the findings of this study are available on request from the corresponding author.
